# Diagnostic Challenges in an Elderly Patient With Cognitive Decline and an Incidental Brain Mass

**DOI:** 10.7759/cureus.82987

**Published:** 2025-04-25

**Authors:** Adi Ahmed, George Okwuasaba

**Affiliations:** 1 Emergency Medicine, Scarborough General Hospital, Scarborough, GBR; 2 Geriatrics, Scarborough General Hospital, Scarborough, GBR

**Keywords:** brain tumor, cns lymphoma, dementia, differential diagnosis, glioblastoma, mri head, neuroimaging, neuro-oncology, primary brain tumors, primary cns lymphoma

## Abstract

Cognitive impairment in the elderly is often attributed to neurodegenerative processes such as dementia or delirium. However, in some cases, structural brain lesions, including malignancies like glioblastoma and lymphomas, can present similarly. This case report is of a 79-year-old male with progressive cognitive decline who was initially suspected to have dementia but was later found to have a brain mass. The absence of focal neurological deficits and a normal initial CT scan delayed the recognition of an underlying malignancy. An MRI ultimately revealed a lesion concerning glioblastoma or primary central nervous system lymphoma (PCNSL). Given the patient’s frailty, the neuro-oncology multidisciplinary team (MDT) recommended best supportive care rather than an invasive biopsy.

This case underscores the importance of thorough assessment and neuroimaging in cases of unexplained cognitive decline and highlights the diagnostic pitfalls that can lead to misdiagnosis.

## Introduction

Cognitive decline is frequently encountered in the elderly population, with dementia being one of the most common etiologies. Alzheimer’s disease and vascular dementia account for the majority of cases; however, other conditions, including reversible causes such as metabolic disturbances, medication side effects, and structural brain lesions, must be considered. The challenge lies in differentiating primary neurodegenerative processes from other causes that may be treatable or require different management strategies [1].

Brain tumors, particularly glioblastoma and primary central nervous system lymphomas (PCNSL), can present subtly, often mimicking symptoms of dementia [2]. This diagnostic overlap can result in the misattribution of symptoms to age-related cognitive decline, delaying appropriate intervention. This case illustrates the difficulties in distinguishing neurodegenerative disease from neoplastic processes and highlights the importance of advanced neuroimaging when clinical suspicion remains high [3,4].

## Case presentation

A 79-year-old male with a history of hypertension, hypothyroidism, and rheumatoid arthritis was brought to the hospital by his family due to concerns about his progressive confusion over two months. He exhibited increasing forgetfulness, disorientation, and episodes of wandering, leading to safety concerns.

Prior to symptom onset, the patient was independent with all activities of daily living (ADLs), including medication management, meal preparation, and personal care. He lived alone, was socially active, and drove short distances. His rheumatoid arthritis was mild and managed with non-steroidal anti-inflammatories (NSAIDs). His general practitioner had initially referred him to a memory clinic, suspecting a new diagnosis of dementia.

Three weeks after the referral was made, the patient presented to the emergency department with ongoing cognitive impairment. On initial assessment in the emergency department, the patient was alert but confused, with a Glasgow Coma Scale (GCS) score of 14/15. Clinical observations were within normal range, with a temperature of 36.3, blood pressure of 139/85 mmHg, heart rate of 50, respiratory rate of 16, and oxygen saturation of 94% on room air.

**Figure 1:**
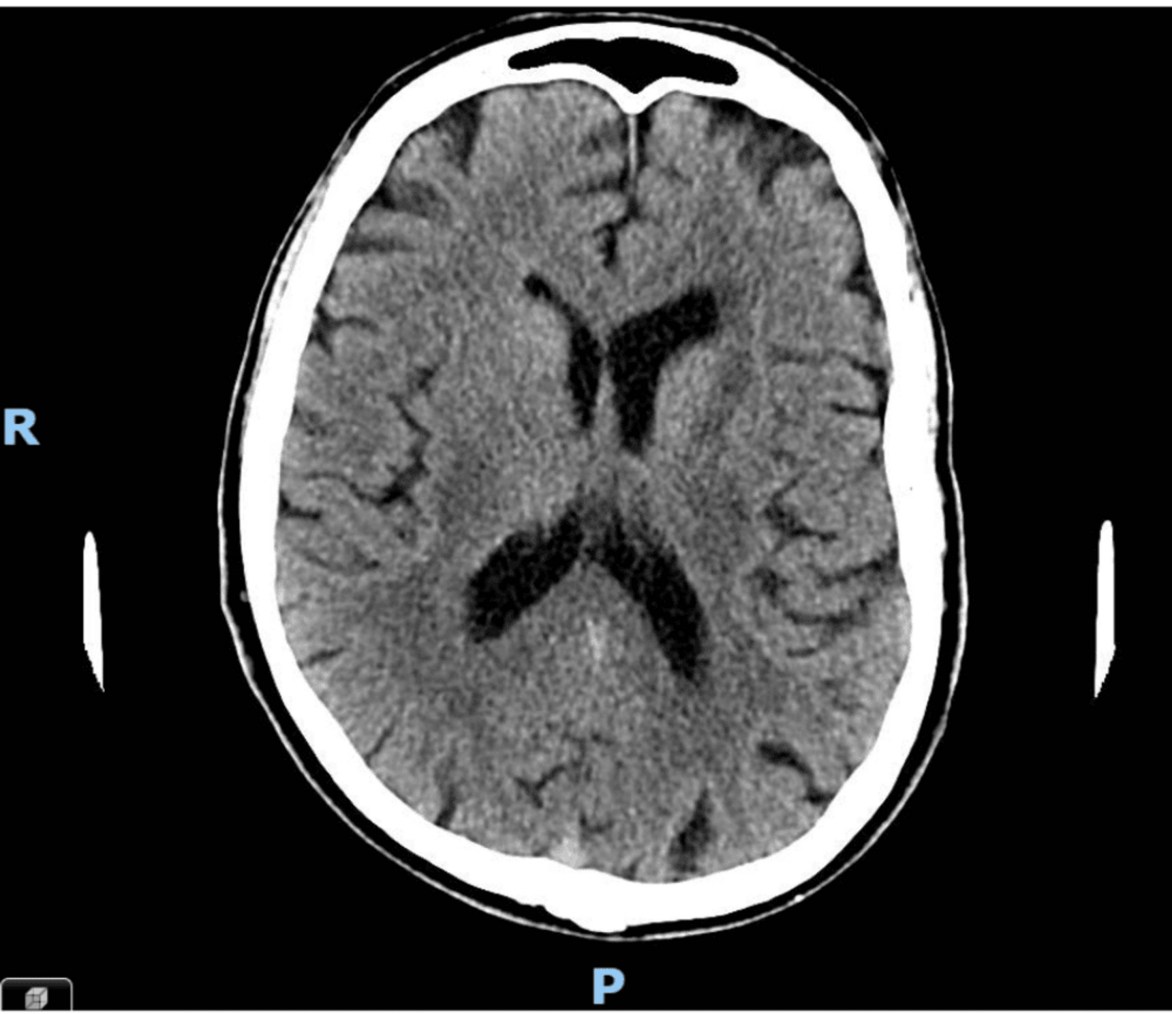
Non-contrast CT head No evidence of intracranial hemorrhage, acute infarction, or obvious space-occupying lesion was noted at the time of reporting. Ventricles and CSF cisterns were initially reported as normal. However, retrospective review in light of subsequent MRI findings revealed subtle asymmetry between the lateral ventricles, suggestive of early right-sided mass effect.

Physical examination was unremarkable, with no focal neurological deficits noted. Blood tests, including B12, folate, thyroid function, and electrolytes, were all within normal limits. A computed tomography (CT) head scan performed on admission showed no acute abnormalities, supporting the likelihood of a cognitive impairment syndrome rather than an acute neurological event (Figure 1).

**Figure 2:**
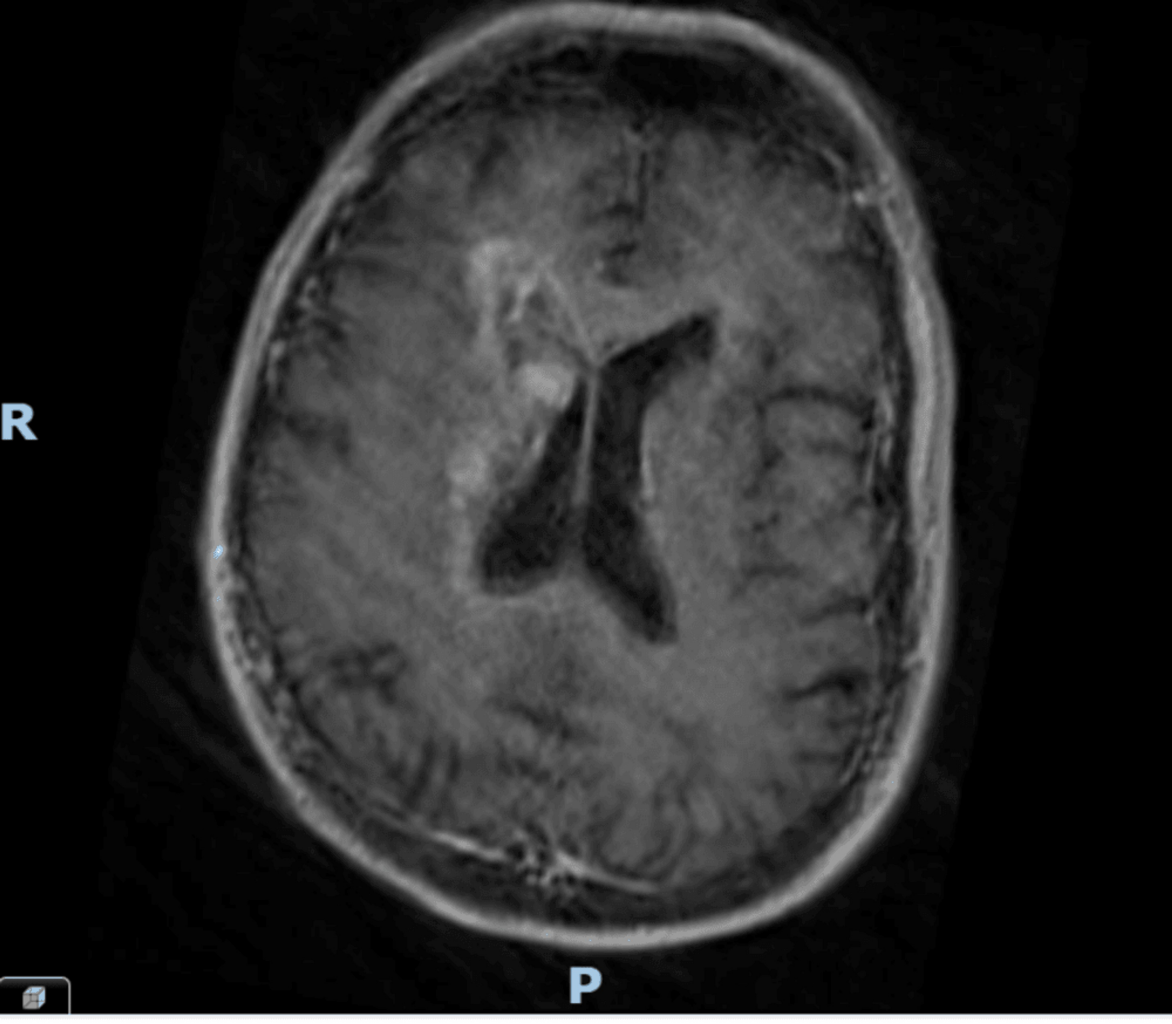
Brain MRI (T1) with contrast Artefactual study due to motion. Abnormal signal and swelling involving the right-sided corpus striatum, internal capsule, thalamus, and periventricular white matter are demonstrated.

**Figure 3:**
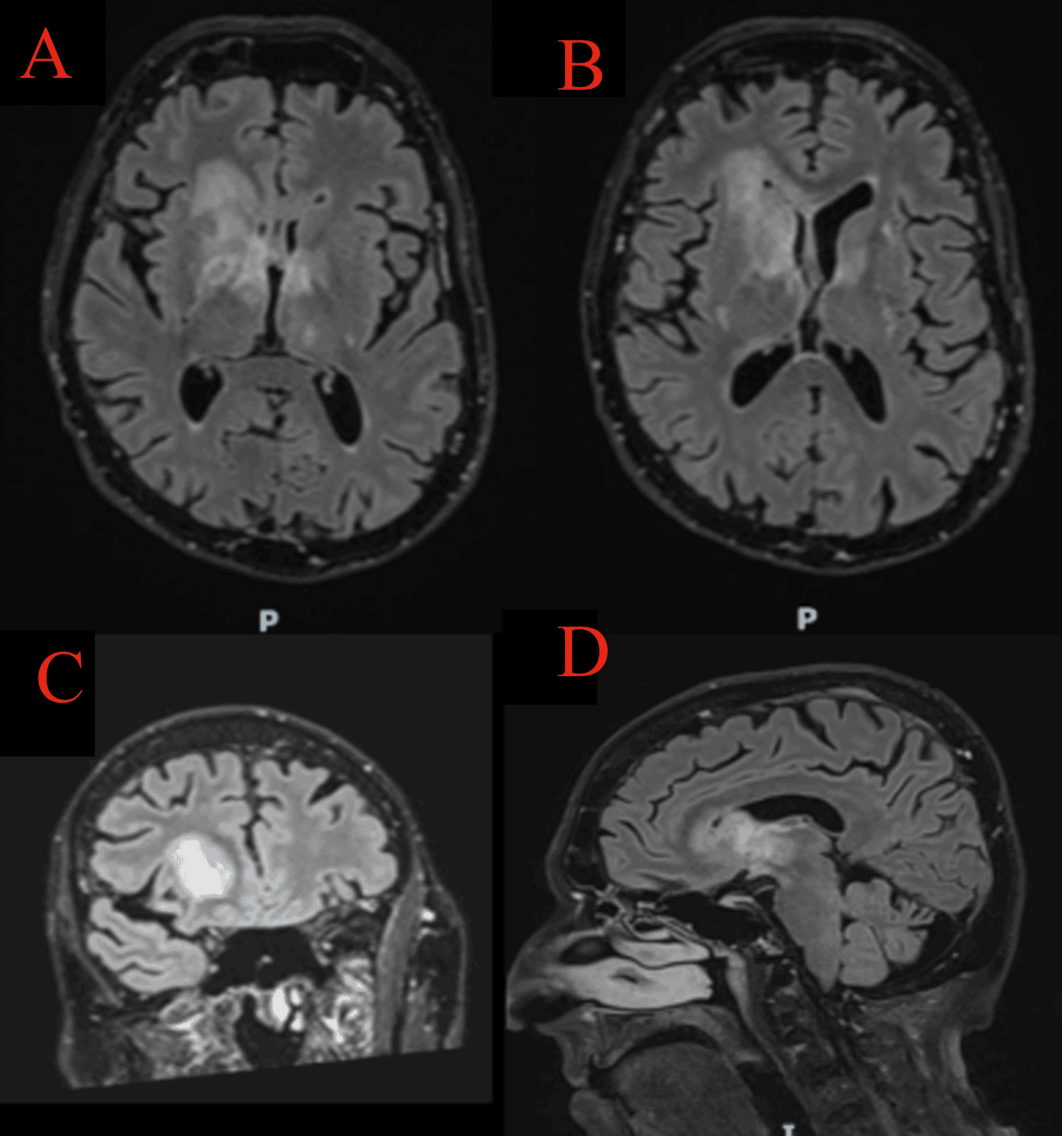
Brain MRI (FLAIR) Brain MRI scans of FLAIR demonstrated multiple high-intensity regions. Axial views (A, B) reveal hyperintensities in the right corpus striatum, extending to the frontal periventricular region, anterior thalamus, corticospinal tract, and splenium of the corpus callosum. Coronal imaging (C) highlights further involvement of the left anterior thalamus, raising concern for contralateral disease. The sagittal view (D) delineates the extent of corpus callosal involvement. There is no evidence of hemorrhage, hydrocephalus, or acute infarction. These findings suggest differentials of high-grade glioma or primary central nervous system lymphoma (PCNSL). FLAIR: fluid-attenuated inversion recovery

Despite conservative management, the patient’s condition continued to deteriorate; however, further collateral history revealed a recent history of visual disturbances, dizziness, and bradycardia, raising concern for an alternative diagnosis beyond primary dementia. A magnetic resonance imaging (MRI) scan with contrast of the brain was subsequently performed two weeks after his initial presentation. This revealed a large, irregularly enhancing lesion involving the right basal ganglia, internal capsule, thalamus, and corpus callosum (Figures 2-3). The differential diagnoses included glioblastoma and PCNSL. A CT scan of the thorax, abdomen, and pelvis showed no evidence of systemic malignancy.

A neuro-oncology multidisciplinary team (MDT) meeting comprising consultants in neurology, neurosurgery, oncology, and radiology was convened to review the case. Given the patient’s age, functional decline, significant frailty, and poor performance status, the team concluded that a brain biopsy would be high risk and unlikely to meaningfully alter the overall prognosis or management. The patient lacked the capacity to participate in decision-making at this stage; however, his two sons, who held health and welfare lasting power of attorney, were fully informed of the clinical findings, potential risks of further investigation, and the likely prognosis of 3-6 months. Following a best interests discussion, the family expressed agreement with the decision to focus on comfort measures and were satisfied with the clear, compassionate care plan.

The best supportive care was initiated, including regular paracetamol and low-dose morphine sulfate for pain, with haloperidol available as needed for agitation. The patient was discharged home with anticipatory medications and a completed Recommended Summary Plan for Emergency Care and Treatment (ReSPECT) form outlining a ceiling of care, including a do not attempt cardiopulmonary resuscitation (DNACPR) decision and treatment of reversible conditions where appropriate.

## Discussion

Diagnostic pitfalls in cognitive decline

This case exemplifies the potential for misdiagnosis when an elderly patient presents with cognitive impairment without overt neurological deficits. The initial working diagnosis of dementia was reasonable given the patient’s age and symptoms. However, certain red flags, such as the relatively rapid progression, visual disturbances, and fluctuating mental status, prompted reconsideration of the initial diagnosis.

A common diagnostic pitfall in evaluating cognitive decline is the over-reliance on CT imaging. While a CT head scan is useful for ruling out acute hemorrhages or large masses, it lacks the sensitivity to detect early neoplastic processes, such as PCNSL. In this case, although the initial CT scan was reported as normal, a retrospective review revealed subtle asymmetry between the lateral ventricles - an early radiological clue that was not appreciated at the time. This highlights the importance of revisiting initial imaging in light of clinical deterioration and the need for heightened vigilance when evaluating atypical presentations.

An MRI with contrast is the gold standard for evaluating unexplained cognitive impairment and should be considered early when the diagnosis is unclear [5,6].

Differentiating glioblastoma from PCNSL

Glioblastoma and PCNSL are both high-grade brain tumors that can present with cognitive decline, mimicking dementia. However, they have distinct imaging characteristics. Glioblastomas typically appear as lesions with necrotic cores and ring enhancement on MRI, predominantly affecting the cerebral hemispheres. In contrast, PCNSL usually presents as homogeneously enhancing deep periventricular lesions without necrosis. In this case, the MRI findings suggested both possibilities, but a definitive diagnosis could not be established without a biopsy.

Earlier use of MRI may have expedited the diagnostic process and, had the patient been referred prior to significant functional decline, may have enabled a biopsy and consideration of treatments such as high-dose methotrexate or corticosteroids in the case of PCNSL. While prognosis in elderly patients with these diagnoses remains guarded, earlier intervention could have modestly improved quality of life or provided an opportunity for short-term disease-modifying therapy.

Emerging imaging techniques, such as advanced diffusion-weighted imaging and MR spectroscopy, may provide non-invasive methods to distinguish between these conditions more accurately [3,7,8].

The role of MDT and ethical considerations

This case highlights the ethical and practical considerations in the management of frail elderly patients with suspected malignancy. The neuro-oncology MDT weighed the risks of an invasive biopsy against the benefits of establishing a definitive diagnosis. In this scenario, aggressive intervention would likely not have altered the overall prognosis, reinforcing the principle of patient-centered care.

Shared decision-making played a crucial role in determining the appropriate course of action, balancing diagnostic certainty with quality-of-life considerations. The family was supported in understanding the implications of the diagnosis and the rationale for palliative management.

## Conclusions

In summary, this case highlights the challenges in distinguishing neurodegenerative diseases from neoplastic processes in elderly patients presenting with cognitive decline. Cognitive impairment should not be automatically attributed to dementia, as atypical features warrant further investigation. While CT scans may fail to detect subtle neoplastic lesions, MRI with contrast should be considered in unexplained cases to improve diagnostic accuracy. Additionally, decision-making in frail patients should prioritize the quality of life, with MDT discussions playing a crucial role in guiding appropriate management.
